# Use of a Technology-Based Fall Prevention Program With Visual Feedback in the Setting of Early Geriatric Rehabilitation: Controlled and Nonrandomized Study

**DOI:** 10.2196/66692

**Published:** 2025-02-11

**Authors:** Carolin Steinmetz, Christina Stenzel, Maj Sylvester, Denis Glage, Anne Linke, Monika Sadlonova, Christine A F von Arnim, Marlena Schnieder, Miroslava Valentová, Stephanie Heinemann

**Affiliations:** 1 Department of Geriatrics, University Medical Center Göttingen, Robert-Koch-Straße 40, Göttingen, 37075, Germany, +49 551 39 68248; 2 Institute of Sports Science, Georg-August-University Göttingen, Göttingen, Germany; 3 Department of Psychosomatic Medicine and Psychotherapy, University Medical Center Göttingen, Göttingen, Germany; 4 Department of Cardiovascular and Thoracic Surgery, University Medical Center Göttingen, Göttingen, Germany; 5 German Center for Cardiovascular Research (DZHK), partner site Lower Saxony, Göttingen, Germany; 6 Department of Neurology, University Medical Center Göttingen, Göttingen, Germany

**Keywords:** fall prevention, fall prevention program, early geriatric rehabilitation, gerontology, older adult, elder, aging, digital exercise intervention, digital activity, physical exercise, functional capacity, new technology, technology-based, digital intervention, feasibility

## Abstract

**Background:**

The Otago program (OP) is evidence-based and focuses on fall prevention in older people. The feasibility and usability of a short-term digital program modeled after the principles of the OP in the setting of early geriatric rehabilitation (EGR) are unclear.

**Objective:**

This study investigated the feasibility and usability of an additional technology-based fall prevention program (FPP) in the setting of EGR.

**Methods:**

We performed a feasibility study in the setting of EGR. A sample of 30 patients (mobility at least by walker; mini-mental status test score >17) was recruited between March and June 2024 and compared with a retrospective cohort (n=30, former EGR patients). All patients in the intervention group (IG) received a supervised, OP-modified FPP thrice/week for 20 minutes using a technology-based platform called “Pixformance.” The device is a digital trainer and enables real-time corrections. The primary end point was the feasibility (given when 80% of the IG participated in 6 trainings within 2 weeks). Secondary outcomes were usability (patients’ and facilitators’ perspective; ≥75%), risk of falls (Berg Balance Scale), mobility (Timed Up and Go Test), functional independence (Functional Independence Measure), and activities of daily living (Barthel Index). Several further exploratory end points were analyzed including anxiety and depression (Four-Item Patient Health Questionnaire; PH-Q4). Data were accessed at entry to EGR and after 2 weeks prior to discharge. To analyze the pre-posttest results, the dependent Student *t* test and the Wilcoxon test were applied. A mixed ANOVA with repeated measurements was used for statistical analyses of time-, group-, and interaction-related changes.

**Results:**

A cohort of 60 patients (mean 80.2, SD 6.1 y; 58% females, 35/60) was analyzed. The main indication for EGR was stroke (9/60, 15%). Patients were recruited into a prospective IG (n=30) and a retrospective control group (n=30). Of the 30 patients in the prospective IG, 11 patients (37%) completed 6 training sessions within 2 weeks. Reasons why participants did not complete 6 training sessions were diagnostic appointments (33%), pain/discomfort (33%), or fatigue (17%). EGR patients rated FPP usability at 84% and facilitators at 65% out of 100%. Pre-posttest analysis of the standard assessments showed a significant interaction in Berg Balance Scale (<.01). In both groups, a significant improvement over time was found in the Timed Up and Go Test (<.01), Barthel Index (<.01), and Functional Independence Measure (<.01). Likewise, in the IG, the PH-Q4 score (.02) improved.

**Conclusions:**

While the technology-based FPP in the EGR setting was generally well-accepted by patients, with high usability ratings, its feasibility was limited. Only 37% of participants completed the required additional training sessions. Further studies should test the technology-based FPP as an integrated part of the EGR complex therapy concept. Our findings suggest potential benefits of incorporating technology-based FPPs in EGR, but further refinement is needed to enhance participation and feasibility.

## Introduction

### Hospitalization in Old Age and Its Consequences

In 2050, around 22.4 million people in Germany will be ≥65 years old. It is predicted that the number of hospital stays in this cohort will increase by around 30% over the next 6 years [1].

In the case of acute medical illness with hospitalization, old and multimorbid patients have a high prevalence of hospital-associated disability [2] with impairment of activities of daily living, cognition, malnutrition, and sensory deficits [3]. Functional impairments such as muscle atrophy of the lower extremities and balance disorders following hospitalization are very common (30%‐80%) [4,5]. The consequences of this deterioration include rehospitalization, nursing home admissions [6], an increased number of falls, a poorer quality of life (QoL), increased consumption of health-related resources [7], and an increase in mortality [8]. Preventing hospital-associated disability therefore is crucial because it significantly impacts patient outcomes and overall health care systems.

### Early Geriatric Rehabilitation

Early geriatric rehabilitation (EGR) is a specialized multidisciplinary and multiprofessional approach for older patients in Germany who need rehabilitation treatment due to acute health events such as injuries or surgeries that require hospitalization [9]. In general, the duration of an EGR is 2 weeks and comprises 20 training sessions in acute medical care. The main aim of EGR is to improve functional independence as well as QoL so that patients can be released to their homes, an inpatient rehabilitation program, or lower-level care such as long-term nursing care [10].

### Fall Prevention Program

Fall prevention programs (FPPs) are often used to optimize functional limitations in older people. The Otago program (OP) is one of these programs; it is evidence-based and was explicitly developed for older people. Long-term studies have shown significant results with regard to the reduction of falls and fall-related injuries [11,12], an increase in strength and balance as well as an increase in confidence in being able to continue with everyday activities [13]. The OP showed the greatest effectiveness in the high-risk groups of over 80-year-olds and people with a history of falls [13,14]. We hypothesized that such prevention programs are not only effective as long-term interventions but can also be effective when the duration of the intervention is short. Previous studies in the area of prehabilitation have shown that older, multimorbid patients benefit from short-term interventions of 5‐14 days [15,16].

Likewise, Martínez-Velilla et al [17] conducted a randomized, controlled intervention study in 370 older patients (age 87.3, SD 4.9 y) who were hospitalized for acute treatment. The intervention group (IG) included individualized moderate strength training, balance exercises, and gait training. The control group (CG) received standard treatment and interventions when needed. The training was safe and 2 sessions daily were sufficient to significantly improve mobility measured by short physical performance battery (SPPB) and the Barthel Index (BI) during an average hospital stay of 5 days, whereas the CG showed a deterioration in mobility [17]. In particular, assessments for measuring a patient’s mobility (eg, Timed Up and Go Test [TUG] or SPPB) have the highest sensitivity in the EGR setting compared with other methods and are suitable to determine the effects of an FPP [17,18].

### Digital Interventions

The number of digital interventions in geriatrics is increasing and may help to improve outcomes such as physical activity or balance. A systematic review of eHealth applications in geriatric rehabilitation showed that digital interventions are promising and have the potential to improve rehabilitation outcomes. In 16 out of 17 studies, eHealth applications were feasible in this setting [19].

The technology-based device “Pixformance” is a digital trainer that uses an integrated 2D full high-definition camera to scan 25 body points, enabling real-time corrections. The screen is divided into 2 parts, one part acts like a mirror and the other shows a person who is demonstrating the exercise. It has been shown that videos that show real people in action are effective in achieving long-term behavioral change [20].

However, acceptance of digital interventions in older persons is often limited by factors such as unfamiliarity with technology, cognitive decline, physical impairments, and concerns about privacy or security. On the other hand, there might be several challenges when it comes to implementing digital interventions for older adults from a facilitators’ perspective. Facilitators are individuals who assist patients in initiating the digital FPP and remain present while the patient interacts with the device. This study is the first to use “Pixformance” in the EGR setting as part of an evidence-based FPP to investigate feasibility and usability.

The purpose of this study is to evaluate the feasibility and usability (patients’ and facilitators’ perspectives) of a short-term technology-based FPP in the setting of EGR, performed in addition to the standard complex therapy concept. Furthermore, the effect of the FPP on the risk of falls, functional independence, activities of daily living, mobility, and cognitive performance compared with a retrospective CG was analyzed in order to determine which assessments can be used to sensitively detect changes due to an additional FPP. This analysis will be used to make informed decisions about the study design and instruments used in a follow-up project. The pre-posttest of muscle status, QoL, anxiety and depression as well as frailty status, which are assessed only in the IG, will also provide information about the sensitivity of these instruments for detecting change in this older patient cohort.

## Methods

### Patient Population

Patient recruitment was carried out in the Department of Geriatrics at the University Medical Center Göttingen, Germany, between March and June 2024.

All patients undergoing an EGR complex therapy, at least able to walk with a walker, and cognitively fit to slightly cognitively impaired (mini mental status test score >17) were eligible for inclusion.

Patients were excluded if they were unable to understand the study information and give written consent due to poor German language skills, or had cognitive or visual impairments.

### Study Setting

**Figure 1.**
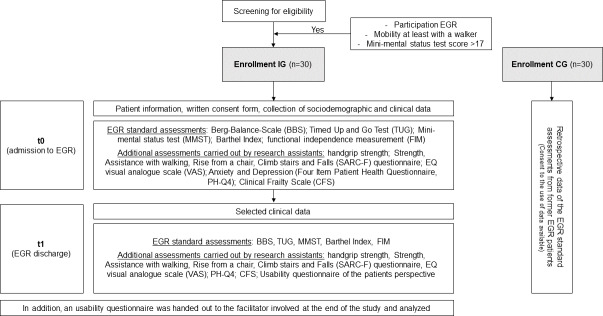
Research design and conducted assessments. CG: control group; EGR: early geriatric rehabilitation; IG: intervention group.

This is a prospective 2-arm feasibility study in the setting of EGR. The IG was recruited prospectively and the CG retrospectively. Eligible patients for the IG were asked to participate in the feasibility study by physicians at the beginning of EGR during regular clinical consultations. All patients were provided study information and gave their written informed consent. Subsequently, the baseline assessment and follow-up assessment took place (Figure 1).

A retrospective CG consists of former patients who have undergone EGR complex therapy at the same department in the last 3 months prior to the recruitment of the prospective group and fulfill the inclusion criteria specified in this feasibility study. All included patients gave written consent for the use of their pseudonymized data for scientific purposes upon admission to the University Medical Center Göttingen. All CG patients were screened and included in the CG according to their order of admission.

### Sample Size

A sample size of 60 patients (IG: n=30; CG: n=30) has 80% power to detect a standardized difference in means of 0.74 with 80% power, using a 2-group *t* test with a 5% 2-sided significance level. This corresponds to comparably large effects, which are likely to be clinically relevant. The sample size was seen as feasible, taking into account the number of potentially eligible patients undergoing EGR within 3 months, as well as the inclusion and exclusion criteria. The study design is comparable to a recently conducted pilot study by Fränzel et al [21] which includes a FPP with 58 participants (IG: n=29; CG: n=29) in the same setting. This study found significant developments in patient mobility measured by TUG, SPPB, and gait speed [21].

### Procedure

**Figure 2.**
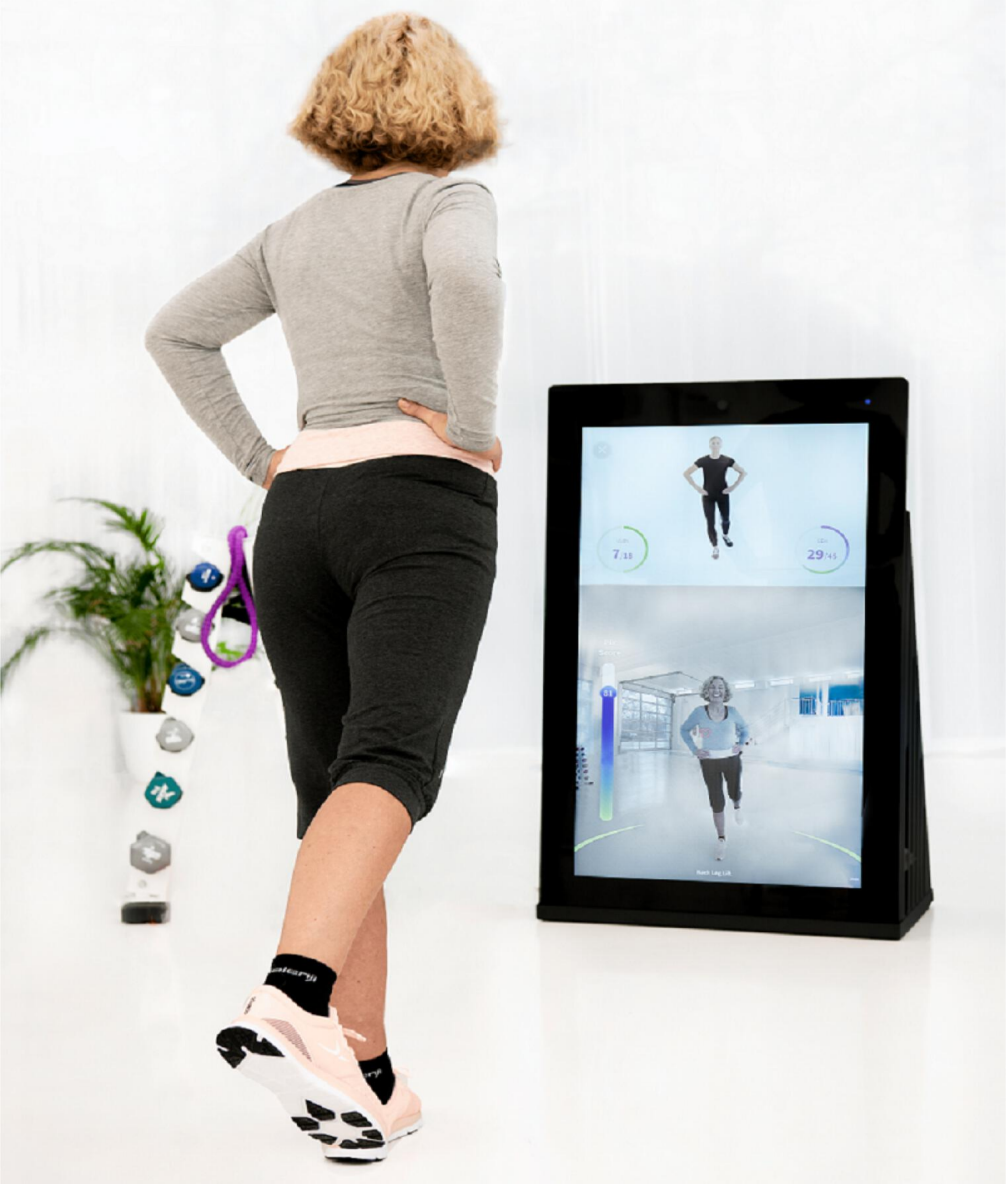
The pixformance station (picture provided by pixformance sports GmbH).

All patients of the IG received the regular EGR complex therapy concept, which consists of 20 sessions, including regular physiotherapy, occupational and speech therapy as well as psychological support. In addition, 6 sessions of a supervised FPP modified according to the OP [22] using the technology-based platform called “Pixformance” (Figure 2) were offered to IG participants 3 times per week.

The content of the 2-week training program is summarized in Table 1.

**Table 1.** Overview of the content of the fall prevention program.

Category	Details
Frequency	Thrice/week; 10 repetitions, 1 set
Intensity	Body weight, Rating of Perceived Exertion scale 13‐15 (medium)
Time	20 min
Type	mobilization, coordination, strengthening exercises focusing on the lower extremities, relaxation/stretching
Exercises	Examples include shoulder and torso rotation, calf raises, lateral leg lift, standing hamstring curl, foot lift and grinding heel in sitting position, and standing up and sitting down from a chair

Before being discharged from the EGR, all participants in the IG received an Otago booklet to continue the exercises at home.

### Outcomes

The primary end point was the feasibility of the technology-based FPP in the setting of EGR. We defined feasibility as at least 80% of the patients completing 6 training sessions within 2 weeks in addition to the regular 20 therapeutic sessions in the EGR. For the statistical analysis, we report how many participants completed the training as well as the reasons for not completing the training in absolute numbers and percentages.

### User Experience

Usability was assessed from the patients’ and facilitators’ perspectives. Facilitators did not provide therapy directly; instead, they addressed technical issues, such as starting, operating, or stopping the device. All training instructions and feedback were delivered by the device itself, not by the facilitators. In this study, facilitators included both geriatric staff (2 physical therapists and 2 occupational therapists) and study personnel (3 sport science students and 1 medical student), all of whom received training in device operation. All facilitators provided written informed consent.

At the end of the study, all patients and facilitators filled out a usability questionnaire. The results of the structured questionnaire were quantitatively analyzed. The device was considered to be usable if the “Overall rating for the training with the device” and “Overall rating for using the device” was ≥75%, modified according to Rabinovich et al [23]. For the statistical analysis, we report the results of the usability questionnaire as the percent of the 2 highest values on the 5-point Likert scale.

### Secondary Outcomes

In addition to feasibility and user experience data, further end points are detailed in Figure 1. Other secondary outcomes included the risk of falls assessed by Berg Balance Scale [24], mobility assessed with the TUG [25], functional independence carried out with the help of the Functional Independence Measure (FIM) [26], and activity of daily living collected by BI [27,28].

Data were assessed at the start of the EGR and after 2 weeks. How the additional technology-based FPP influences the effect of EGR complex therapy was examined using standard assessment data from the IG and a retrospective CG.

### Statistical Analysis

We used the Kolmogorov–Smirnov test for the assessment of normal distributions. Continuous and categorical variables are presented by mean (SD) with absolute and relative frequencies, respectively. Two-group comparisons of baseline variables were performed using Student’s *t* test and chi-square test of independence for continuous and categorical variables. To analyze the results of the pre-posttest, dependent Student *t* test and the Wilcoxon signed rank test were applied. A 2-way mixed ANOVA with repeated measurements was used for statistical analyses of time- (t0/t1), group- (IG or CG), and interaction-related (between time and group) changes. In all analyses, a *P*<.05 was considered statistically significant. The results of the usability questionnaire include the percent of the 2 highest values on the 5-point Likert scale. Analyses were performed using the IBM Statistical Package for the Social Sciences (SPSS version 29.0; IBM Co).

### Ethical Considerations

This feasibility study was approved by the local ethics committee of the University Medical Center Göttingen (application number: 22/1/24; January 23, 2024) and conforms to the Declaration of Helsinki. All participants signed informed consent forms.

## Results

### Study Population

A total of 70 patients were screened for eligibility for the prospective IG. Of these, 40 were excluded because of “no interest” (n=13), “poor mobility” (n=10), “hospital stay too short” (n=5), “low cognition” (n=4), “low cognition and poor mobility” (n=3), “visual impairment” (n=2), and “others” (n=3). In the end, 30 patients from the IG participated in the FPP.

A sample of 60 patients (mean age 80.2 years, SD 6.1 years; 58% females, 35/60; IG: n=30; CG: n=30) were included in the analysis. Patients of the prospective IG were consecutively recruited (n=30; mean age 79.0 years, SD 6.0 years; 53% female, 16/30) at admission to EGR. The retrospective CG (mean age 85.0 years, SD 4.6 years; 63% female, 19/30) is made up of former EGR patients who were treated in the 3 months prior to the “Pixformance” intervention and fulfilled all inclusion and exclusion criteria.

The baseline characteristics of the IG showed the cohort to be significantly younger and taller than the CG. With regards to clinical and medical history characteristics, patients in the IG were more likely to be taking a higher number of medications and had a degree of disability, a post-Covid condition, and depressive symptoms in comparison to the retrospective CG (Table 2).

**Table 2.** Baseline characteristics of the cohort.

Characteristics	All (n=60)	IG^a^ (n=30)	CG^b^ (n=30)	*P* value
Age (years), mean (SD)	82.0 (6.1)	79.0 (6.0)	85.0 (4.6)	<.01^c^
Sex, n (%)				
Male	24 (40)	14 (47)	10 (33)	.34^d^
Female	35 (58)	16 (53)	19 (63)	.34^d^
Unknown	1 (2)	0 (0)	1 (2)	.31^d^
Height (cm), mean (SD)	166.4 (8.4)	168.6 (8.0)	164.1 (8.4)	.04^c^
Weight (kg), mean (SD)	71.4 (16.6)	73.8 (18.3)	68.9 (14.6)	.26^c^
BMI (kg/m²), mean (SD)	25.8 (5.2)	26.1 (5.9)	25.5 (4.5)	.67^c^
Blood pressure (mmHg^e^), mean (SD)				
Systolic	N/A^f^	131.7 (23.1)	N/A	N/A
Diastolic	N/A	73.1 (12.0)	N/A	N/A
Heart rate (beats/min), mean (SD)	N/A	71.4 (11.3)	N/A	N/A
Number of medications (regularly), mean (SD)	7.6 (4.1)	9.1 (3.9)	6.1 (3.7)	.01^c^
Need for nursing assistance, n (%)	30 (50)	16 (53)	14 (47)	.87^d^
Any degree of disability, n (%)	9 (15)	8 (27)	1 (3)	<.01^d^
Situation of living, n (%)				
Living alone	N/A	11 (18)	N/A	N/A
Visual aid, n (%)	41(68)	24 (80)	17 (57)	.07^d^
Hearing aid device, n (%)	16 (27)	7 (23)	9 (30)	.08^d^
Walking aid, n (%)	34 (57)	15 (50)	19 (63)	.29^d^
Walker	26 (43)	12 (40)	14 (47)	.60^d^
Cane	4 (7)	2 (7)	2 (7)	1.0^d^
Forearm crutch	5 (8)	1 (3)	4 (13)	.16^d^
Main diagnosis for EGR^g^ admission, n (%)				
Poststroke or suspected stroke	9 (15)	5 (17)	4 (13)	.72^d^
Cardiac decompensation	8 (13)	4 (13)	4 (13)	1.0^d^
Acute kidney failure	4 (7)	2 (7)	2 (7)	>.99^d^
Vertigo including syncope	4 (7)	3 (10)	1 (3)	.30^d^
Concomitant diseases, n (%)				
Diabetes mellitus	23 (38)	12 (40)	11 (37)	.12^d^
Hypertension	35 (58)	16 (53)	19 (63)	.06^d^
Dyslipidemia	15 (25)	6 (20)	9 (30)	.39^d^
Stroke or TIA^h^	12 (20)	6 (20)	6 (20)	.37^d^
Dementia	7 (12)	3 (10)	4 (13)	.41^d^
Kidney failure	13 (42)	5 (17)	8 (27)	.19^d^
Cancer disease<5 years	5 (8)	3 (10)	2 (7)	.54^d^
Cancer disease ≥5 years	5 (8)	2 (7)	3 (10)	.50^d^
Thyroid disorder	8 (13)	6 (20)	2 (7)	.21^d^
Rheumatism	10 (17)	8 (27)	2 (7)	.08^d^
Condition after COVID-19 infection	20 (33)	17 (57)	3 (10)	<.01^d^
Depression	5 (8)	1 (3)	4 (13)	.04^d^
Anxiety	5 (8)	3 (10)	2 (7)	.19^d^

a IG: intervention group.

b CG: control group.

c Independent *t* test.

d Chi-square test.

e mmHg: millimeters of mercury.

f N/A: not available.

g EGR: early geriatric rehabilitation.

h TIA: transient ischemic attack.

### Feasibility of the FPP

Out of a total of 30 patients in the IG, 11 (37%) participated in 6 trainings within 2 weeks. Of these 11 patients, 7 (64%) completed the FPP including all strengthening/balance exercises. The remaining 4 patients ended their training session early or skipped one or more important strengthening or balance exercises.

The main reasons why participants did not complete the amount of training were the inability to attend the FPP due to diagnostic appointments (33%), pain/discomfort (33%), or fatigue (17%).

### User Experience

The analysis of user experiences from the patients’ and facilitators’ perspectives is summarized in Table 3. The 8 facilitators were on average 35.1 (SD 12.3) years old. A quarter of the facilitators were male (2/8, 25%) and 3 quarters were female (6/8, 75%). The group members had an average work experience of 20.0 (SD 8.8) years.

**Table 3.** Overview of the results of the user experience questionnaire from the perspective of patients and facilitators.

Usability items	Rating	Patients’ perspective (n=26), (%)	Facilitators’ perspective (n=8), (%)
Instructions by facilitators for using the device were clear	Agreeing	96	—^a^
There were technical problems	Never	85	—
Training with the device was comfortable	Often	92	—
Training with the device was embarrassing	Never	100	—
Corrections during the training by the device were clear	Agreeing	89	—
Willingness to continue the learned exercises at home	≥3 months	54	—
Overall patient usability rating (%)(100%=verygood experience), mean (SD)	—	84.0 (15.90)	—
Instructions by the manufacturer for using the device were clear	Agreeing	—	50
Difficulties when commissioning the device	No starting problems	—	63
Regular use of the device was easy	Agreeing	—	50
The exercise selection of the device was easy to handle	Agreeing	—	63
Selected exercises could be easily adapted during the training	Agreeing	—	25
The device was easy to switch on and off	That worked very well	—	75
I am in favor of the future use of the device	Yes, very	—	50
Overall facilitator usability rating (%) (100%=verygood experience), mean (SD)	—	—	65.4 (20.0)

a Not applicable.

The overall usability rating for the device from the patients’ perspective was 84.0% (SD 15.9%), where 0% means a bad experience and 100% a very good experience. The facilitators rated the usability of the device at 65.4% (SD 20.0%).

### Secondary Outcomes

The results of the EGR standard assessments are indicated in Table 4. For 22 patients in the IG and 29 patients in the CG, complete measurements at both time periods were recorded. The 2-way mixed ANOVA showed a significant interaction between groups over time in the Berg Balance Scale (<.01). Likewise, a significant positive development over time was seen in both groups in TUG time (<.01), FIM motor subscale (<.01) score, and BI (<.01).

**Table 4.** Descriptive and 2-way mixed ANOVA results for the early geriatric rehabilitation routine assessments at baseline (t0, admission) and after 2 weeks (t1, discharge) differentiated by group.

Parameters	CG^a^, mean (SD)	IG^b^, mean (SD)	*P* value		
Time	Group	Interaction
Berg Balance Scale (score)			.54	.02	<.01
t0^c^	31.3 (12.5)	33.6 (10.0)			
t1^d^	38.4 (8.7)	24.5 (7.5)
Timed Up and Go Test (s)			<.01	.40	.82
t0	22.1 (9.0)	20.2 (7.6)			
t1	17.5 (7.5)	16.1 (6.7)
Minimental status test (score)			.07	.21	.21
t0	24.2 (3.3)	24.6 (4.3)			
t1	24.7 (3.0)	27.2 (3.9)
Barthel Index			<.01	.68	.78
t0	61.6 (17.5)	63.7 (18.2)			
t1	76.2 (12.9)	76.8 (15.9)
FIM^e^ motor subscale (score)			<.01	.35	.64
t0	53.8 (12.2)	51.0 (10.6)			
t1	67.0 (10.9)	65.3 (6.6)

a CG: control group.

b IG: intervention group.

c t0: baseline (early geriatric rehabilitation admission).

d t1: after 2 weeks (early geriatric rehabilitation discharge).

e FIM: Functional Independence Measure.

The Wilcoxon signed rank test confirmed the significant development of the mixed ANOVA in Berg Balance Scale (BBS) in IG (.01; n_paired_results_ =22) and CG (<.01; n_paired_results_ =29). In the IG, 5 patients improved and 17 showed poorer results in BBS between baseline and EGR discharge, whereas in the CG, 17 participants improved, 2 showed poorer results and 10 did not change in BBS.

Table 5 summarizes the results of muscle status, QoL, anxiety and depression, and frailty status, which were additionally assessed by study personnel in the IG. A significant improvement in Four-Item Patient Health Questionnaire (PH-Q4) score (.024) from baseline to EGR discharge was observed. Furthermore, the frailty status and the positive screen for sarcopenia each decreased by 27%.

**Table 5.** Pre-posttest and descriptive results for the assessments at baseline (t0) and after 2 weeks of early geriatric rehabilitation (t1) of the intervention group (IG).

Parameters	IG		*P* value
	t0 (n=30)	t1 (n=26)	
Handgrip strength (kg), mean (SD)	24.9 (7.6)	24.7 (7.3)	.93^a^
Female	26.2 (6.4)	20.0 (6.2)	.40^a^
Male	23.9 (8.6)	28.4 (6.1)	.18^a^
CFS^b^ (score), mean (SD)	4.3 (1.5)	3.8 (1.6)	.27^a^
Frailty (CFS ≥4), n (%)	21 (70)	13 (43)	>.99^c^
SARC-F^d^ score, mean (SD)	4.0 (2.4)	3.4 (1.9)	.28^a^
Sarcopenia (positive screen SARC-F ≥4), n (%)	18 (60)	10 (33)	.13^c^
EQ VAS^e^, mean (SD)	52.1 (15.4)	60.4 (13.0)	.06^a^
PH-Q4^f^, mean (SD)	3.3 (2.4)	1.6 (1.9)	.02^a^

a Dependent *t* test.

b CFS: Clinical Frailty Scale.

c Chi-square test.

d SARC-F: Strength, Assistance with walking, Rise from a chair, Climb stairs and Falls questionnaire.

e EQ VAS: European Quality of Life Visual Analogue Scale.

f PH-Q4: Four-Item Patient Health Questionnaire.

## Discussion

### Summary of Target and Findings

In this study, we aimed to evaluate the feasibility and usability of a short-term technology-based FPP in the EGR setting from the patients’ and facilitators’ perspectives. The FPP was conducted in addition to the conventional complex therapy concept. Furthermore, the effect on the risk of falls, daily function, mobility, and cognitive performance compared with a retrospective CG was analyzed. In the IG, the pre-posttest of muscle status, QoL, anxiety and depression as well as frailty status were additionally examined. Regarding the primary end point, we observed the limited feasibility of the FPP in the EGR setting, while usability and acceptance from the patients’ perspective were proven. The results of the BBS showed a significant decrease in the IG and a significant improvement in the CG. Further significant improvements from EGR admission to discharge were observed in TUG, FIM, BI, and PH-Q4.

This study is the first to use the “Pixformance” device in the EGR setting as part of an evidence-based FPP.

### Study Cohort

The number of older patients receiving complex therapy in EGR in Germany (211,270 participants in 2007 to 274,926 in 2020) is increasing steadily due to demographic change [29]. The baseline characteristic of our cohort is similar to others who performed studies in the EGR setting [21,30,31]. The other studies reported an average age of 78 to 87 years, the admission of more women than men, and strokes, cardiac decompensation, and hip fractures as the most common admission diagnoses [21,30,31]. In our cohort, significant differences between the groups were observed in age, height, number of regularly taken medications, degree of disability, condition after COVID-19 infection, and depression. Our CG is an unmatched-pairing retrospective comparison cohort of former patients from the time point directly before the intervention. The patients were consecutively included in the CG, beginning at the start of the intervention and going backward in time until 30 patients were identified. In this feasibility study, a CG was included in order to determine which assessments might be used to sensitively detect changes due to an additional FPP in order to design a follow-up project.

Participants of the IG were younger and had more often severe concomitant diseases. The high number of patients after COVID-19 infection in the IG may explain why only 11 out of 30 patients completed 6 training sessions within 2 weeks. Fatigue is one of the most common reasons why the FPP was not completed. Especially older adults aged 65 years or older are at greater risk of persisting symptoms associated with COVID-19; fatigue is 1 of the 5 common long-term symptoms [32].

### eHealth in Geriatrics

Kraaijkamp et al [19] published a systematic review of eHealth in the setting of geriatric rehabilitation. eHealth is defined as “the use of digital information and communication to support and/or improve health and healthcare” [19,33]. The authors summarized that eHealth is often feasible and can potentially improve geriatric rehabilitation outcomes. Simple eHealth interventions are more likely to be feasible for older patients receiving geriatric rehabilitation [19]. “Pixformance” can be seen as such a “simple” eHealth intervention. Likewise, the authors concluded that there is a lack of evidence on usability which might hamper the implementation [19].

### Feasibility of the FPP

In our study, feasibility was defined as participation in 6 FPP training sessions throughout the EGR. The most common reason for missing a training session was due to medical examinations outside the ward. Sometimes, there were delays in the morning so patients could not participate in the FPP. The clinical routine always took priority. The low participation rate was therefore due to logistical problems that the study team was unable to solve. An additional training session in the EGR setting therefore seems to be too much. Similarly, Fränzen et al [21] conducted a pilot study including a FPP in the form of a square-stepping exercise (SSE) in the EGR setting and investigated its feasibility. The required minimum number of SSE units, each lasting 30 minutes, was 6 during the EGR stay. The amount of exercise units is in line with our target and definition of feasibility. The SSE program proved to be feasible. The main difference between the 2 interventions was that the SSE was performed as a part of conventional physiotherapy included in the complex therapy concept [21]. In our feasibility study, the FPP took place in the afternoons in addition to the complex therapy concept. In a future study, we will include the study intervention as part of conventional physiotherapy in the complex therapy concept so that logistical problems do not affect the intervention.

### User Experience

We have investigated the usability of the technology-based FPP and followed the advice from Kraaijkamp et al [19] to pave the way for a possible implementation [19]. User experience was assessed independently from the facilitators’ and patients’ perspectives. Usability from the patients’ perspective was achieved, while the facilitators’ did not reach the target of ≥75%. The results are surprising in that eHealth is often not sufficiently tailored to age-related barriers such as cognition, physical ability, perception, and motivation [34,35]. “Pixformance” as a “simple” eHealth intervention seems to be attractive to patients. Similarly, a usability study of technology-based exercise games in older patients following hip replacement surgery confirmed that the cohort enjoyed performing exercises under digital guidance [36]. In contrast to this, usability issues were pointed out by facilitators.’ Only every second respondent was in favor of using the device in the future in the EGR setting. The item “selected exercises could be easily adapted during the training” was only agreed on by 25% of the facilitators. The low rating could be because the exercises had to be changed via a separate computer platform. Facilitators’ explained that there was not enough time to learn how to adapt the exercises and navigate the training platform in detail. This may be due to the nature of the study, which was conducted in addition to the staff’s normal daily activities [37].

### Secondary Outcomes

The BBS is widely used in clinical settings to assess the risk of falls, particularly in older individuals with balance impairments or in geriatric wards within general hospitals [38,39]. A score of less than 45 on the BBS is considered the threshold for increased fall risk. A change of 7 points is usually considered clinically relevant. When the score lies between 35 and 44 points, an increased risk of falls is indicated [40]. At baseline, the mean BBS scores of our cohort ranged from 31 to 34 points, indicating an increased risk of falls. Interestingly, the results of the BBS showed a significant decrease in the IG (−9.1 points) while the CG showed a significant improvement (+7.1). Even so, both groups remained in the range of increased fall risk. One reason for this unexpected outcome could be that the BBS may not be the most appropriate assessment tool to capture changes resulting from a FPP. The BBS primarily includes tasks that were not trained in the FPP, such as “Retrieving object from floor” or “turning to look behind.” For a follow-up project, the SPPB appears to be a more suitable assessment tool. As mentioned in the introduction, measuring a patient’s mobility using tools like the SPPB has shown the highest sensitivity in EGR settings compared with other methods [17,18]. The SPPB consists of 3 parts: “gait speed test,” “chair rising test,” and “balance test.” These tests are well suited for measuring changes resulting from the FPP [41]. Moreover, the SPPB is frequently used in studies conducted in cohorts similar to ours [21,42]. It should be noted, however, that there was some missing data in the IG, resulting in only 22 paired results being included in the analysis. Therefore, these findings should be interpreted with caution.

Further significant positive developments (ie, the patients’ condition improved after 2 weeks) based on the multimodal and interdisciplinary EGR complex therapy concept [9,10] were found in functional independence (FIM), activity of daily living (BI), mobility (TUG), and anxiety and depression (PH-Q4). These instruments, therefore, seem appropriate for measuring differences due to digital FPP in further studies.

### Limitations

This feasibility study has a relatively small cohort and includes an unmatched-pairing retrospective comparison cohort. Further studies with larger cohorts and a randomized controlled design with blinded assessors are needed to validate the results presented. Key factors such as disability (yes or no), sex (male or female), and age (younger than 81 years vs 81 years and older) should be considered in the 1:1 block randomization process with random block lengths to ensure a homogeneous cohort at baseline. Additionally, the training intensity of the FPP might be increased by incorporating a digital balance pad or components of the SSE unit intervention [21] to improve outcomes.

### Conclusion

Due to the high percentages of frail and sarcopenic patients admitted to EGR with an elevated fall risk, there is a significant unmet need for a FPP. However, the feasibility of the technology-based FPP was not achieved, as only 37% of participants completed 6 additional FPP sessions within 2 weeks, primarily due to diagnostic appointments, pain/discomfort, or fatigue. Usability analysis indicates that EGR patients accept the technology-based FPP, while staff members are less convinced. Significant positive changes from EGR entry to discharge were observed in TUG, FIM motor subscale, and BI, independent of the FPP. A statistically significant, but not clinically relevant improvement in the BBS was noted in the CG, while the IG showed a significant positive change in the PHQ-4 score, indicating fewer symptoms of anxiety and depression. Further studies should explore the technology-based FPP as an integrated part of the EGR complex therapy concept, increase the FPP intensity by incorporating a digital balance pad or components of the SSE, and assess progress from admission to discharge using the SPPB instead of the BBS.

## Abbreviations

BBS: Berg Balance Scale
BI: Barthel Index
CG: control group
EGR: early geriatric rehabilitation
FIM: Functional Independence Measure
FPP: fall prevention program
IG: intervention group
OP: Otago program
PH-Q4: Four-Item Patient Health Questionnaire
QoL: quality of life
SPPB: short physical performance battery
SSE: square-stepping exercise
TUG: Timed Up and Go Test
